# HCC biomarkers – state of the old and outlook to future promising biomarkers and their potential in everyday clinical practice

**DOI:** 10.3389/fonc.2022.1016952

**Published:** 2022-11-28

**Authors:** Sophie Schlosser, Deniz Tümen, Barbara Volz, Katja Neumeyer, Niklas Egler, Claudia Kunst, Hauke Christian Tews, Stephan Schmid, Arne Kandulski, Martina Müller, Karsten Gülow

**Affiliations:** Department of Internal Medicine I, Gastroenterology, Hepatology, Endocrinology, Rheumatology, and Infectious Diseases, University Hospital Regensburg, Regensburg, Germany

**Keywords:** hepatocellular carcinoma, biomarker, personalized medicine, alpha-fetoprotein, liquid biopsy, circulating tumor cells, circulating nucleic acids, exosomes

## Abstract

Hepatocellular carcinoma (HCC) is one of the most common and deadly tumors worldwide. Management of HCC depends on reliable biomarkers for screening, diagnosis, and monitoring of the disease, as well as predicting response towards therapy and safety. To date, imaging has been the established standard technique in the diagnosis and follow-up of HCC. However, imaging techniques have their limitations, especially in the early detection of HCC. Therefore, there is an urgent need for reliable, non/minimal invasive biomarkers. To date, alpha-fetoprotein (AFP) is the only serum biomarker used in clinical practice for the management of HCC. However, AFP is of relatively rather low quality in terms of specificity and sensitivity. Liquid biopsies as a source for biomarkers have become the focus of clinical research. Our review highlights alternative biomarkers derived from liquid biopsies, including circulating tumor cells, proteins, circulating nucleic acids, and exosomes, and their potential for clinical application. Using defined combinations of different biomarkers will open new perspectives for diagnosing, treating, and monitoring HCC.

## 1 Hepatocellular carcinoma – a high disease burden

Hepatocellular carcinoma (HCC) is one of the most frequent tumor types worldwide. Its clinical importance has been rising tremendously in the past two decades: The incidence increased about 75% (1). Concurrently, HCC is ranked 5th most common tumor worldwide (2). In 2020 905,677 new cases of liver cancer worldwide were diagnosed. About 80% of them were HCC (2).

Regarding death caused by cancer, HCC ranks 3rd place (2). The World Health Organization expects more than one million deaths due to liver cancer by 2030 (3). Men are almost three times more likely to be affected than women.

73% of HCC cases occur in Asia (2). The reason for this geographic variance is the endemic occurrence of hepatitis viruses, the most common risk factor for HCC. Despite the decrease in the number of Hepatitis B virus (HBV) infections due to the global introduction of vaccination programs, one-third of all HCC cases worldwide are still based on chronic HBV infection (33%) (1, 4). Hepatitis C (HCV) infection is the second most important risk factor. Even after the introduction of new, highly efficient combination therapies with polymerase inhibitors, protease inhibitors, and non-structural protein 5A (NS5A) inhibitors in 2013, more than one-fifth of HCCs are due to HCV infection (1). Another relevant risk factor with a substantial global disease burden and potential to grow is the metabolic syndrome. Since 1975, overweight and obesity have tripled worldwide (5). Other risk factors are alcohol-related liver cirrhosis (30%), primary biliary cholangitis, primary sclerosing cholangitis, hemochromatosis, and alpha1-antitrypsin deficiency (1). Aflatoxin is a mycotoxin that can also trigger HCC and is mainly found in nuts, dried fruits, and spices.

Physicians should regularly monitor the risk groups depicted above for the occurrence of HCC. Given the high burden of disease, there is a tremendous need for valid and cost-effective biomarkers that can

- identify individual risks for developing HCC,
- detect HCC in an early stage,
- predict therapy response to specific therapies,
- monitor response to therapy and predict adverse side effects of cancer therapies,
- and predict cancer recurrence.

These biomarkers should be easy to obtain and easy to analyze routinely.

## 2 What characterizes an optimal biomarker?

**Figure 1:**
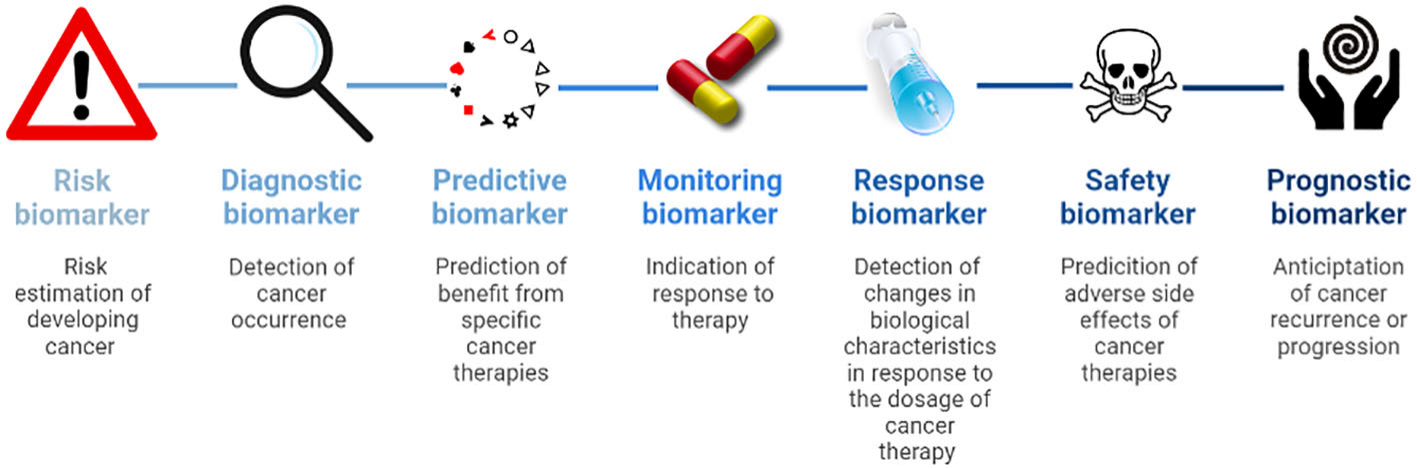
Overview of biomarker types.

Modern medicine depends on reliable biomarkers for screening, diagnosis, disease monitoring, prognosis, predicting therapy success, response to therapy, and treatment safety. The BEST (Biomarkers, EndpointS, and other Tools) glossary of the FDA-NIH Biomarker Working Group defines biomarkers as a characteristic that is measured as an indicator of normal biological processes, pathogenic processes or biological responses to an exposure or intervention, including therapeutic interventions.” (6). They contain molecular, histologic, radiographic or physiologic characteristics.” (6, 7). The FDA-NIH Biomarker Working Group distinguishes between (6):

- Susceptibility or risk biomarkers are associated with the chance of developing cancer and are essential to define risk populations for surveillance.
- Diagnostic biomarkers detect cancer occurrence.
- Predictive biomarkers identify patients that might benefit from specific cancer therapies.
- Monitoring biomarkers indicate the activity of the disease and response to therapy.
- Pharmacodynamics or response biomarkers show changes in biological characteristics in response to the dosage of cancer therapy.
- Safety biomarkers predict adverse side effects of cancer therapies.
- Prognostic biomarkers anticipate cancer recurrence or progression (**Figure 1**).

For clinical applicability, biomarkers must meet the following criteria:

- The biomarker has to be valid, reliable, and objective.
- The sample collection must only involve a minimal risk for the patient.
- The biomarker has to be easy to collect.
- The sample must be stable under clinical and laboratory conditions.
- Samples should be able to be analyzed on a routine basis.
- Analysis has to be feasible and rapid.

## 3 State of the old – conventional biomarkers recommended by guidelines

Unfortunately, there are only a few biomarkers for HCC in daily practice so far: alpha-fetoprotein (AFP), HSP70 (HSPA7), glypican 3 (GPC3), and glutamine synthetase (GS). They are not even considered in all major guidelines due to their limited diagnostic quality. In the following, we will briefly review the use of biomarkers for HCC in daily practice:

### 3.1 Susceptibility biomarkers

Up to date, no susceptibility biomarkers are recommended by HCC guidelines and in daily clinical practice for stratifying the risk of HCC occurrence.

### 3.2 Diagnostic biomarkers

AFP is the most established clinical biomarker in clinical practice for detecting HCC. It is a transport protein for copper, nickel, fatty acids, and bilirubin expressed during the embryonic phase in human cells. Serum AFP levels diminish rapidly after birth and remain very low during adulthood (8). Of clinical relevance, in adults, AFP is mainly found in tumor cells of the liver, testes, and ovaries. It has been shown that AFP dampens the immune response and can enhance immunological tolerance toward tumors. In detail, secreted AFP interferes with the maturation and function of dendritic cells, leading to a decreased antigen presentation and induction of immune responses (9, 10). Furthermore, AFP interferes with T cell proliferation and shifts T cell response to a more regulatory phenotype. Thus, AFP promotes the immune system’s tolerance toward the tumor (11, 12).

Although AFP is commonly used as a biomarker for detection, it imposes enormous limitations: AFP has a very low-test sensitivity and specificity. Several studies showed disappointing or even contradictory results (9). AFP expression is absent in around 80% of early HCC (13). In many cases, liver damage also leads to the upregulation of AFP expression and secretion (14, 15). Especially in patients with high viral load, the determination of AFP displays low reliability. Low cut-off levels above 20 ng/ml show high sensitivity but low specificity, whereas high cut-off levels above 200 ng/ml raise specificity but lower sensitivity (16). Thus, serum AFP levels cannot reliably discriminate between chronic liver damage (e.g., fibrosis and cirrhosis) and HCC. Therefore, the Practice Guidance of the American Association for the Study of Liver Diseases (AASLD) and the Pan-Asian adapted European Society for Medical Oncology (ESMO) Clinical Practice Guidelines recommend biannual ultrasound with or even without determination of AFP levels for patients with liver cirrhosis and hepatitis-virus-infected patients (17, 18). Child Pugh-Stage and co-morbidities should allow curative or palliative therapy. The European Association for the Study of the Liver (EASL) even explicitly opposes the determination of AFP for the reasons given above (19). Nevertheless, according to new meta-analyses, the combination of ultrasound and AFP is a reliable diagnostic approach to detect HCC (7, 20). Zhang et al. recommended 400 ng/ml AFP protein in terms of sensitivity and specificity, whether AFP is used alone or combined with ultrasound (21).

As for AFP, the EASL guidelines assess other conventional diagnostic biomarkers, AFP-L3 and Des-gamma-carboxy-prothrombin (DCP), as unsuitable for cost-efficiency reasons. Nonetheless, studies of last years revealed that a combination of the clinical markers gender and age with the biomarkers AFP-L3, AFP, and DCP, is a good diagnostic marker (22). The combination is named the GALAD score after the initials of the biomarkers.

AFP-L3, an isoform of AFP, binds the lectin lens culinaris agglutinin (LCA) and is produced by malignant hepatocytes (23). A meta-analysis has shown that AFP-L3 has high specificity but low sensitivity for the diagnosis of early-stage HCC, suggesting that AFP-L3 is more valuable for ruling out HCC in conditions with elevated AFP levels than for diagnosing early HCC (24).

DCP is a defective prothrombin and results from a lack of post-translational carboxylation of the prothrombin precursor in HCC cells. Most large-scale studies have been performed in patients mainly of HCV- or HCB-related etiology (25–28).

Johnson et al. developed the score initially (22). In their study, the performance of the GALAD model was significantly better than the simple combination of AFP-3, AFP, and DCP alone. In a prospective phase 3 cohort study by Tayob et al. of 50 HCC patients and 484 controls, the GALAD score was associated with a substantial improvement in sensitivity for detecting HCC. However, in this study, a limitation of the GALAD score also shows up since the advantage of increased sensitivity was offset by a high number of false-positive results (29). In contrast, the study of Best et al. showed a high specificity of 93.3% (30) and was reliable in the subgroup of HCV and NAFLD patients. In another case-control study from Germany and a pilot cohort study in Japan, the GALAD score has been shown to detect early-stage HCC with high accuracy in patients with NASH, with and without cirrhosis (30–32) and thus could facilitate the monitoring of patients with NASH. In addition, another study shows that the GALAD score is also excellent for distinguishing HCC from chronic liver disease in an HCV subgroup of a cohort of Chinese patients (33). Therefore, despite limitations, the GALAD score seems one of the best options for HCC detection and will likely find its way into the guidelines.

If the diagnosis of HCC cannot be confirmed by typical contrast agent behavior in imaging, the EASL guidelines recommend a biopsy (19), with subsequent staining of HSP70, Glypican-3 (GPC3), and glutamine synthetase (GS).

HSP70 is a chaperone involved in protein folding, protein translocation, and regulation of transcription. In contrast to normal cells, many tumor entities, including HCC, overexpress HSP70 and secrete it into the extracellular matrix. Expression profiling identified HSP70 as a molecular marker for the detection of early HCC (34, 35). In addition, HSP70 serum levels enable discrimination between chronic hepatitis, cirrhosis, and HCC. However, this observation is limited by analyzing a relatively small cohort (86 healthy donors, 50 donors with chronic hepatitis, and 47 HCC patients) (36), and HSP70 is secreted by other tumors as well.

GPC3 belongs to a family of glycosylphosphatidylinositol-anchored cell surface heparin-sulfate proteoglycans. Sung et al. showed that GPC3 is upregulated in HCC tissue and plays an important role in the proliferation of malignant cells (37). Hippo et al. demonstrated that, compared to AFP, GCP3 is a more reliable marker to distinguish between patients with small, well-differentiated HCC and liver cirrhosis (38). However, a meta-analysis by Xu et al. revealed that GCP3 is inferior to AFP in the differential diagnosis between HCC and liver cirrhosis (39). Indeed, GCP3 expression displays no correlation to the expression of AFP.

The enzyme GS catalyzes the synthesis of glutamine, the primary energy source of tumor cells, from glutamate and ammonia in the liver. GS’s mRNA, protein, and activity were increasingly upregulated in precancerous lesions to advanced HCC (40, 41). GS displays a 50-59% sensitivity and specificity of 86-90% (42, 43).

A combination of the three markers, GCP3, the chaperone HSP70, and glutamine synthase for early detection of HCC revealed a sensitivity of 72% and a specificity of 100% (42). Thus, the combination of multiple markers results in advanced sensitivity and specificity.

### 3.3 Predictive biomarkers

Predictive biomarkers play almost no role in the choice of HCC therapy. There are two exceptions:

The curative treatment options for HCC are resection, local ablation procedures, and liver transplantation. Due to the pervasive organ shortage, bridging therapy to transplantation is required, and patients must be carefully selected. According to national guidelines, liver transplantation should not be considered if AFP levels are above 1000 ng/ml due to poor postoperative prognosis (18, 44–46).

Palliative treatment options for advanced-stage HCC include immunotherapies (atezolizumab/bevacizumab, tremelimumab/durvalumab, nivolumab/ipilimumab, pembrolizumab) and protein tyrosine kinase inhibitors (sorafenib, lenvatinib, donafenib, regorafenib, cabozantinib, apatinib). Ramucirumab is a recombinant human monoclonal antibody that inhibits vascular endothelial growth factor receptor (VEGFR) and is approved for second-line therapy. The REACH-2 trial demonstrated that ramucirumab improved overall survival compared to placebo in patients with hepatocellular carcinoma and AFP levels of at least 400 ng/ml who had previously been treated with sorafenib (47). Patients with lower AFP levels did not benefit.

### 3.4 Monitoring biomarkers

Imaging techniques primarily assess the response of patients with HCC to local or drug therapies. None of the guidelines recommend specific biomarkers for assessing response to therapies. Bruix et al. analyzed two phase 3 studies of prognostic factors and predictors of the benefit of sorafenib in patients with HCC. AFP was not a predictive biomarker of sorafenib benefit (48). However, a recent meta-analysis showed that post-treatment AFP response was significantly associated with overall and recurrence-free survival (49).

### 3.5 Prognostic biomarkers

Like the assessment of response to treatment, screening for relapse after curative treatment is mainly performed by imaging with MRI, CT, or ultrasound. Imamura et al. showed in a study of 249 patients undergoing hepatectomy that AFP levels above 32 ng/ml indicate relapse (50).

In summary, AFP is the only biomarker used for early detection, prediction, and monitoring of response to treatment and disease recurrence. It has significant limitations in sensitivity and specificity, especially when used alone without combination with other biomarkers. Therefore, there is a great need for other or complementary biomarkers to improve the quality of care for patients with HCC.

## 4 Promising new biomarkers for clinical application

Conventional tissue biopsies are invasive and associated with risks for patients. Sometimes tissue biopsies are impossible or very difficult to perform due to the location of the tumor or the presence of multiple lesions. In contrast, biomarkers from body fluids are a promising tool for diagnosing and monitoring tumor diseases, especially because they are not or only minimally invasive and can therefore be obtained without or with low risk. The idea of liquid biopsy is based on molecular analysis of circulating cells that have been shed from the tumor and products from malignant tissue that have been released into biological fluids, such as the bloodstream. Thus, liquid biopsies provide access to tumor-derived materials, including circulating nucleic acids, proteins, exosomes, and circulating tumor cells (CTCs). However, the differences between these various groups of markers in terms of accessibility, stability, and detection must be considered.

### 4.1 Circulating nucleic acids

Nucleic acids are released into the bloodstream after induction of apoptotic or necrotic cell death of tumor cells. These circulating nucleic acids can be divided into two subgroups: (i) circulating tumor desoxyribonucleic acid (ctDNA) and (ii) cell-free ribonucleic acid (RNA).

#### 4.1.1 Circulating tumor DNA

In 1948 Mandel et al. described for the first time that freely circulating DNA is released from dying cells into the peripheral blood (51). Later Leon et al. observed that circulating DNA appeared more frequently in the serum and plasma of cancer patients (52) and reflected the tumor burden (53). Furthermore, ctDNA provides direct access to molecular key information, including genomic (point mutations or copy number variations [CNV]) as well as epigenetic data (changes in DNA methylation) (54).

Apoptotic and necrotic cells release DNA into the extracellular matrix. This DNA can be detected as circulating cell-free DNA in the blood. Solid tumors often show large necrotic areas due to undersupply of oxygen and glucose. Therefore, various tumors, including HCC, can be detected by an increased level of cell-free DNA. Iizuka et al.’s study highlighted the potential diagnostic value of monitoring the amount of cell-free DNA to detect HCC (55). However, elevated cell-free DNA levels have been observed in multiple cancers. Therefore, the amount of cell-free DNA is not HCC-specific. Nevertheless, combining the determination of the amount of cell-free DNA with the detection of HCC-specific protein biomarkers like AFP results in a sensitivity of 87% and specificity of 100% to detect HCC (56). A recent study presents a novel computer-based prediction model that uses comprehensive fragmentomic profiling of cell-free DNA in plasma for early detection of liver tumors. The model showed excellent performance with a sensitivity of 98.8% and 96.8% specificity in detecting primary liver cancer (sensitivity for HCC 96.2% and intrahepatic cholangiocarcinoma 100%). In addition, early-stage primary liver cancer detection was 95.9% (stage I) and 97.9% (stage II). For tumors less than 3 cm in size, the sensitivity was 98.2% (57).

The use of modern technologies such as next-generation sequencing (NGS) allows the detection of mutations in DNA isolated from the blood (58). For example, tumor-specific point mutations of various genes have been detected in the ctDNA of HCC patients. NGS analyses revealed that seven genes and DNA regions derived from ctDNA harbor the most important mutations associated with poor survival in HCC: (i) TERT promoter, (ii) TP53, (iii) CTNNB1, (iv) AXIN1, (v) JAK1, (vi) EPS15 and (vii) CACNA2D4 (59–67). Several studies have shown that analysis of point mutations in these genes is a valuable tool with a clinically relevant impact on prognosis and early detection of HCC (59, 68, 69).

However, the use of somatic mutations previously detected in primary tumor tissue as biomarkers is limited by their variability and low concentration in plasma (70). Besides detection and analysis of point mutations, CNV can be used as early biomarkers and prognostic parameters for HCC (63–66, 71–78).

For example, a characteristic CNV was found in preresection plasma samples of patients with HCC, whereas this CNV was almost absent in post-resection plasma samples. Thus, CNV is an additional marker for detection and treatment surveillance (79, 80).

Furthermore, genomic alterations and epigenetic modifications (e.g., DNA methylation) were detected in ctDNA (81–85). DNA methylation is a mechanism to regulate gene expression and control DNA stability and DNA-protein interactions. DNA methylation is essential in cancer development, especially in HCC formation (54, 86–88). The methylation status of several tumor suppressor genes correlates with HCC occurrence and progression. These genes include p15 and p16, APC, SPINT2, SFRP1, TFP12, GSTP1, and RASSF1A (86, 89, 90). Moreover, specific hypomethylation of the long interspersed nuclear element-1 (LINE-1) (91), methylation of the insulin-like growth factor-binding protein 7 (IGFBP7) gene (92) and hypomethylation of the promotor region of the transcriptional repressor CTCFL were associated with reduced survival in HCC patients (93).

Therefore, combined determination of (i) the amount of circulating cell-free DNA, (ii) additional biomarkers, and (iii) genetic analysis of ctDNA, including detection of tumor-specific point mutations, CNV, and alteration in methylation patterns, are essential tools for detection and prognosis of HCC. Furthermore, genetic aberrations detected in ctDNA provide molecular information about tumor development and possibly response towards treatment. Recent developments allow the analysis of small amounts of ctDNA. This advance will facilitate analyses of ctDNA in the future.

**Figure 2:**
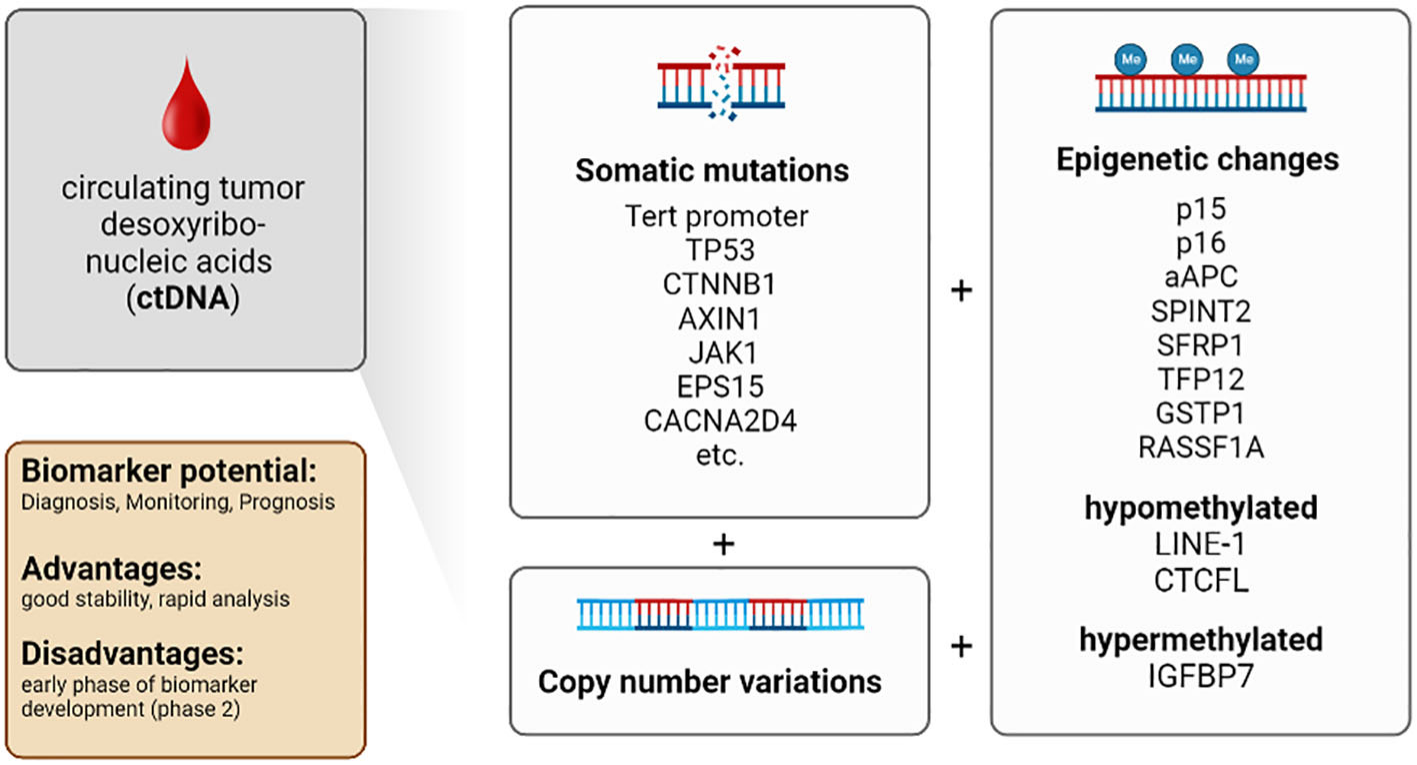
Characteristics of the biomarker class circulating tumor desoxyribonucleic acids (ctDNA) in patients with HCC.

**Conclusion:** ctDNA provides a comprehensive representation of genomic alterations of different tumor regions. Isolation procedures are well-established. Improvements in technology allow for higher sensitivity of analytical assays. The short half-life of ctDNA allows real-time monitoring of cancer development with more accurate clinical correlations. The amounts of blood required for isolation are practical from the clinical point of view. The number of patients and the control groups are also large enough in most studies, and international studies are available in addition to many Asian studies. Most studies with ctDNA are in biomarker development phase 1 or 2 (57, 84, 94, 95). The limitations are that it is difficult to distinguish between ctDNA and circulating free DNA (released from non-malignant cells) and the low concentration of ctDNA in blood. In addition, the short half-life, which on the one hand allows real-time monitoring, is on the other hand a challenge for the analysis and storage of the samples. In comparison to CTCs ctDNA is not suitable for functional assays. Furthermore, analysis is time-consuming and costly, and most emerging assays have not yet been clinically validated (**Table 1**; **Figure 2**).

**Table 1:** Overview of relevant biomarker classes for HCC.

Biomarker	Phase of biomarker development	Biomarker type	Sample	Patient risk	Availability	Stability	Analysis on a routine basis	Rapid analysis
ctDNA	Phase 2	Diagnostic (0.57-0.97)^1^ (96, 97)	Blood Hepatic tissue	Blood: (+) Hepatic tissue: ++	+/-	++	+	++
	Phase 2	Monitoring						
	Phase 2	Prognostic						
circRNA	Phase 3	Diagnostic (0.81-0.89)^1^ (98–101)	Blood Hepatic tissue	Blood: (+) Hepatic tissue: ++	+/-	+/-	+	++
	Phase 2	Monitoring						
	Phase 3	Prognostic						
miRNA	Phase 3	Diagnostic (0.79-0.88)^1^ (102–105)	Blood	(+)	+++	+	+	++
	Phase 3	Prognostic						
lncRNA	Phase 2	Diagnostic (0.88-0.92)^1^ (106–108)	Blood Hepatic tissue	Blood: (+) Hepatic tissue: ++	++	+	+	++
	Phase 2	Prognostic						
Proteins: AFP	Phase 5	Diagnostic (0.61-0.93)^1^ (21, 97)	Blood Hepatic tissue	Blood: (+) Hepatic tissue: ++	+/-	+++	+++	+++
	Phase 5	Monitoring						
	Phase 5	Prognostic						
Exosomes	Phase 2	Diagnostic (n.a.)	Blood	(+)	+++	+++	+	+
	Phase 2	Prognostic						
CTCs	Phase 2	Diagnostic (0.70-0.93)^1^ (97, 109, 110)	Blood	(+)	+++	+++	+	+
	Phase 2	Monitoring						
	Phase 2	Prognostic						

^1^ pooled AUC in systematic reviews.

Relative properties: - limited; + low; ++ medium; +++ high

#### 4.1.2 Cell-free tumor RNA

**Figure 3:**
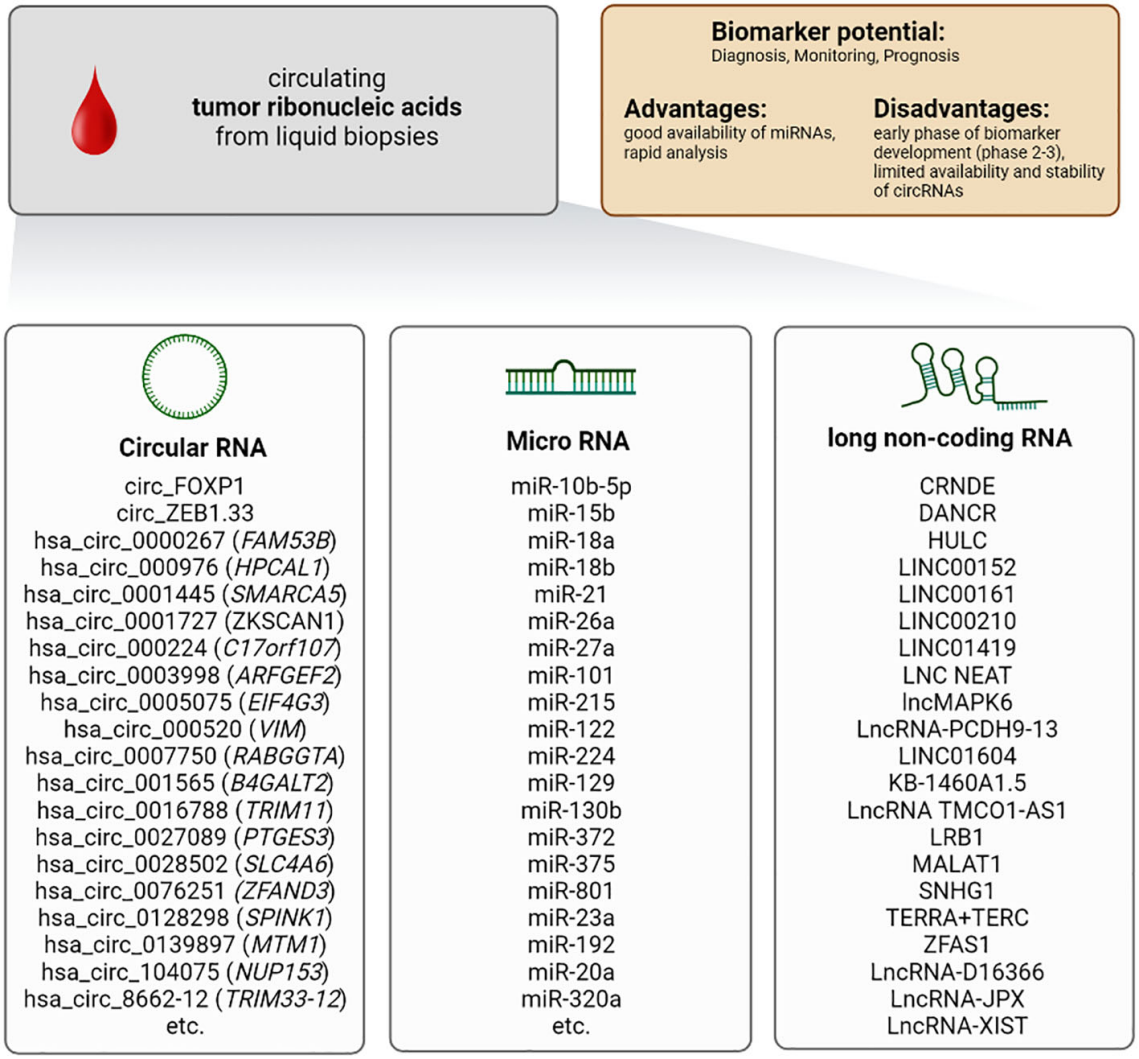
Characteristics of the biomarker class circulating tumor ribonucleic acids (cell-free RNA) in patients with HCC.

In addition to DNA, RNA is also released into the extracellular space. It should be highlighted that RNA is less stable compared to DNA. Therefore, cell-free RNA is associated with proteins, proteolipids, or encapsulated into exosomes preventing its degradation. Three different groups of RNA can be detected in the bloodstream and used as potential biomarkers: circular RNA, micro (mi)RNA, and long-non-coding (lnc)RNA (**Figure 3**).

##### 4.1.2.1 Circular RNA

Circular RNAs are differentially expressed in various cancer tissues (111–114), including HCC (115), and they are closely associated with the initiation and development of cancer. Circular RNAs arise from aberrant by-products or abnormally spliced transcripts (116). Most up-regulated circular RNAs are positively associated with HCC progression, whereas down-regulation usually displays suppressive effects and prevents HCC development. Most data on circular RNA are based on tissue analysis. Therefore, circular RNAs can also be used as markers in normal biopsies. The particular advantage, however, is that these RNAs can also be found in blood and thus be sampled minimally invasively (117, 118).

Several circular mRNAs showed highly interesting results in context with early detection and diagnosis of HCC (115, 119–123). For example, the circular RNAs hsa_circ_001565 (B4GALT2), hsa_circ_000224 (C17orf107), and hsa_circ_000520 (VIM) display a sensitivity of up to 97% and a specificity of up to 92% in the detection of HCC (124). Very similar results were obtained for hsa_circRNA_104075 (NUP153), hsa_circ_0005075 (EIF4G3), hsa_circ_0028502 (SLC24A6) and hsa_circ_0076251 (ZFAND3). They showed comparable sensitivities of up to 96% and specificities of up to 98% (115, 122, 125).

Besides their potential as biomarkers for the early detection of HCC, circular RNAs are of high prognostic value (124, 126–132). Exemplary is a study showing that hsa_circ_0001727 (circZK - SCAN1) expression was positively correlated with HCC prognosis (129). Moreover, the detection of hsa_circ_001565 (B4GALT2), hsa_circ_000224 (C17orf107), and hsa_circ_000520 (VIM) was associated with prolonged relapse-free survival (124). In contrast, low expression of hsa_circRNA8662-12 (TRIM33-12) was closely correlated with poor prognosis (133).

**Conclusion:** Circulating free RNA provides an up-to-date snapshot of the transcriptome. It can indicate cancer and trace it back to its site of origin. Limitations are sample instability and high variability of circulating free RNA expression between different individuals. Therefore, studies aiming to define panels of cell-free circulating RNAs that can be used as general biomarkers for HCC are needed. In addition to the small amount and not yet very detailed purification protocols, the high variability and heterogeneity of cell-free circulating RNA are problematic and currently do not allow for clinical application. In addition, there is always a risk that samples will be contaminated with transcellular mRNA. Unfortunately, there are only very few studies from Europe or the USA. Most of the studies originate from East Asia and are in development phase 3 (121, 123, 131, 134) (**Table 1**). Thus, there is still much development work to be done here.

##### 4.1.2.2 Cell-free micro (mi)RNA

In 2008 Lawrie et al. were the first to describe microRNA (miRNA) as tumor biomarker (135). miRNAs are a member of endogenous non-protein-coding RNA with a size of approximately 20-22 nucleotides. miRNAs are relatively resistant to RNase degradation, boiling, and freeze-thaw cycles (136, 137). miRNAs can be used as markers in tissues and the blood. In terms of patient safety, miRNAs obtained in a liquid biopsy are of particular interest. Especially the stability of these RNAs and their release into the bloodstream makes them attractive as a biomarker. Over the past ten years, miRNAs have become the most intensively studied nucleic acid biomarkers in HCC and have proven valuable in the diagnosis and prognosis of the disease. Nevertheless, many studies have conceptual weaknesses, such as very different non-validated purification methods for miRNAs or unclear sequencing and identification protocols.

miRNAs bind to the corresponding 3´UTR of their target messenger RNA (mRNA) and induce mRNA degradation. Interestingly, there is a correlation between abnormal circulating miRNA levels and pathological characteristics of certain tumors (138–140), including HCC (141). So far, over 70 miRNAs have been proposed as potential biomarkers for HCC (86, 142–159). A meta-analysis evaluated miRNA-21 as a biomarker for early diagnosis of HCC with a sensitivity of up to 88% and a specificity of up to 87% (160). Interestingly, the sensitivity and specificity of the combined miRNA panel miR-29a, miR-29c, miR-133a, miR-143, miR-145, miR-192, and miR-505 were significantly higher than the sensitivity and specificity of the established biomarker AFP regarding detection of small (AUC: 0.833 vs. 0.727) and early-stage HCC (AUC: 0.824 vs. 0.754). Another panel of miRNAs (miR-192, miRNA-125b, and miR-23a) was suitable for predicting the survival time of HCC patients (161). Huang et al. developed in a phase 3 study an HCC risk score consisting of 5 miRNAs (miR-18a, miR-26a, miR-27a, miR-222, miR-223) that correlates with an increased risk of HCC development in cirrhotic patients (162). Thus, miRNA combinations represent a strategy to develop novel diagnostic tools for HCC and improve treatment surveillance.

**Conclusion:** The advantage of using miRNA as a biomarker is its wide range of applications, as miRNAs are involved in many pathogenic processes and have high specificity and reproducibility. miRNAs display inherent stability, and the serum concentration is relatively high. The main challenge in establishing miRNAs as biomarkers is that although the available studies have identified a large number of miRNAs as potential markers, the miRNAs identified vary depending on the specific study. Therefore, a “universal marker” is still lacking. This is due to the lack of standardized protocols for the purification of miRNAs and the fact that the expression of these miRNAs is directly linked to the developmental stage and characteristics of the individual tumor. In addition, comorbidities can lead to an increase of unspecific miRNAs, which interfere with detecting cancer-specific miRNAs. Nevertheless, it should be possible to define miRNA panels that could act as universal markers. These panels can then be used for both prognosis and diagnostics purposes. In studies containing information about the required blood volumes, the required volumes were in a range that could be used in the clinic (≤10ml). Unfortunately, some studies lack this information. Most studies include a relatively large number of participants (>100). The control groups are also sufficiently big in most cases. Interestingly, most recent studies were conducted in East Asia (China) or North Africa (Egypt). However, the number of patients with HCC is increasing in Western countries due to lifestyle and the resulting metabolic syndrome. There is a high heterogeneity of the miRNAs found, and each study finds different miRNAs that can be used as biomarkers. Most studies are in phase 2 (155, 157), and few are in phase 3 (162). We need more studies with clearly defined protocols for the purification of miRNAs and also very precisely characterized cohorts of patients. Therefore, very little can be said about miRNAs as biomarkers for HCC. Generally, they are a potential option that fulfills all the prerequisites for a good marker (**Table 1**).

##### 4.1.2.3 Cell-free long non-coding RNA (lncRNA)

Circular RNA and miRNA are not the only RNAs that can serve as biomarkers for HCC. Furthermore, long non-coding RNA (lncRNA) shifted into the focus as novel potential biomarkers in HCC. Comparable to miRNAs, lncRNAs belong to the group of non-protein-coding RNA transcripts. They exceed a length of 200 nucleotides. Like other non-protein-coding RNAs, lncRNAs can be detected in the blood. Upon isolation, lncRNAs are stable in the plasma (163, 164). They can interact with proteins, DNA, and other types of RNA. They act as modulators of gene expression by regulating transcriptional and post-transcriptional processes and controlling various cellular processes, e.g., genomic imprinting, cell cycle, cell proliferation, differentiation, and apoptosis (165, 166). Various lncRNAs are involved in cancer pathogenesis, controlling invasion, migration, and metastasis of tumor cells (167–169), and several studies showed that dysregulation of lncRNAs promoted the development of HCC (170).

Therefore, lncRNAs might contribute to the panel of beneficial biomarkers in hepatic tumors. One of the first and most studied lncRNAs in HCC is Highly Upregulated in Liver Cancer (HULC). Several studies demonstrated that circulating HULC could be used as a diagnostic marker, being up-regulated in the blood of patients with HCC (171, 172). Another lncRNA of clinical relevance is LINC00152. The amount of LINC00152 in the blood increases from healthy donors to patients with liver cirrhosis and displays the strongest up-regulation in patients with HCC. This close association with progress from liver cirrhosis to HCC underlines LINC00152 abilities as a potential diagnostic biomarker (172, 173). A further significant increase in sensitivity and specificity was achieved by combining HULC with LINC00152 or combining HULC, LINC00152, and AFP (174). However, a more detailed analysis is needed to prove their value as biomarkers for HCC (175).

**Conclusion:** The advantage of using lncRNA as a biomarker is its relatively high stability. lncRNAs are involved in multiple pathological processes and can obtain new insights into the progression of the disease. The disadvantages are similar to those of miRNAs. Since lncRNAs are involved in many physiological and pathophysiological processes, detecting tumor-specific lncRNA is difficult. Interference by co-morbidities cannot be excluded. Some studies directly compare tissue samples and blood samples. However, many studies involve small patient and control cohorts. In addition, most studies have been conducted in East Asia. Care should be taken to include European and American cohorts in the future. Most studies are in biomarker development phase 2 (172, 176–179). Therefore, further detailed studies are needed to improve data on individual lncRNAs and establish defined lncRNA panels that can be used as general markers for detecting HCC. The large heterogeneity of lncRNAs identified in the previous studies, which unfortunately overlap only partially, is the major obstacle to clinical application.

Overall, circulating nucleic acids occur in different forms, e.g., circulating tumor DNA (ctDNA) or cell-free RNA. Although circulating nucleic acids are relatively inert towards degradation, it is still more elaborate to purify, store and analyze them compared to proteins. The advantage of nucleic acids as markers is that they contain essential biological information and may be of prognostic value. But these properties provide a large heterogeneity in terms of expression in tumors of different patients, making clinical application difficult. Interestingly, a combination of nucleic acids and marker proteins displays the highest specificity and sensitivity in HCC diagnosis and could thus help develop new identification panels that can be used universally for detecting HCC. Therefore, combining different biomarkers, regardless of nucleic acid, protein or CTC, might be the most promising approach for the future (**Table 1**).

### 4.2 Proteins

Tumor cells secret various proteins into the extracellular matrix, e.g., proteases required for invasion and metastasis, proteins dampening the immune system, cytokines, growth factors, and angiogenic factors for proliferation. In addition, tumor cells undergo apoptotic or necrotic cell death due to a lack of oxygen and energy. These dying cells release proteins into the bloodstream.

**Figure 4:**
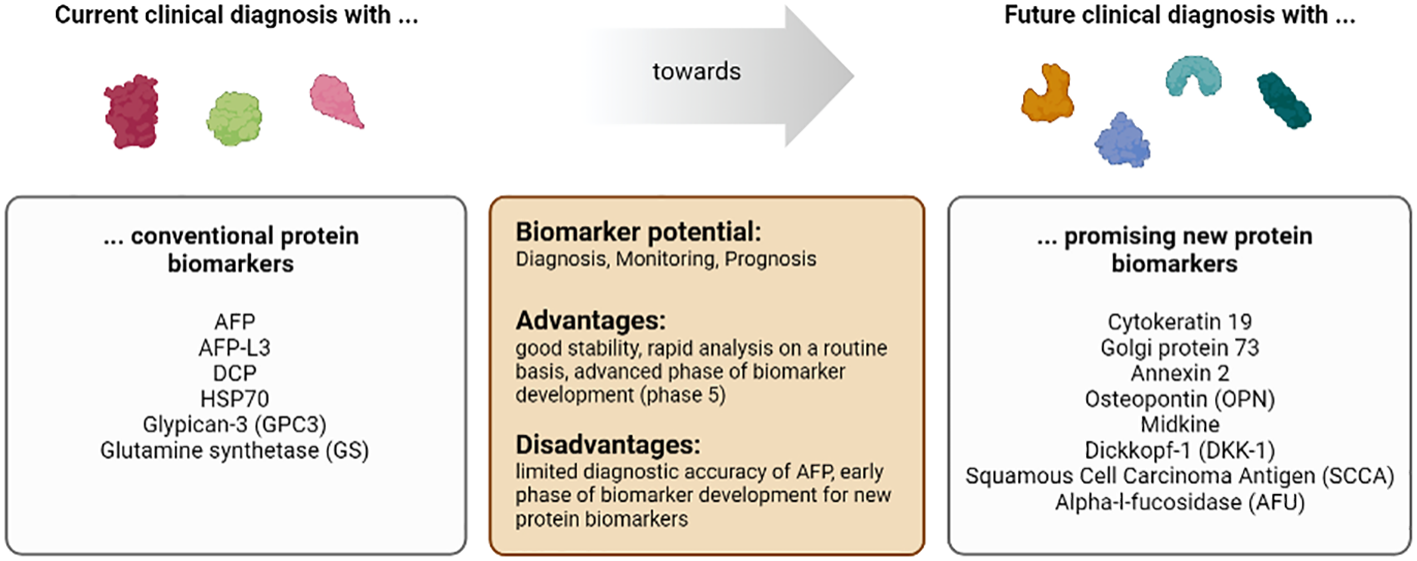
Characteristics of the biomarker class proteins in patients with HCC.

Proteins are very stable in the blood, and there are several methods for identification by immune-linked and biochemical methods. Detecting tumor-specific proteins is less complex and expensive than identifying and purifying cell-free DNAs and RNAs. As described above, proteins are the most commonly used biomarkers in clinical routine (**Figure 4**).

#### 4.2.1 Cytokeratin 19

Cytokeratin 19 belongs to the keratin family. It is a filament protein essential for the structural integrity of cells. Cytokeratin 19 has been associated with poor clinical prognosis in HCC patients in several studies. The co-expression of Cytokeratin 19, AFP, and Glypican-3 is an excellent predictive factor for metastasis and adverse treatment outcomes (180). In addition, concurrent expression of these three proteins was also associated with poor survival (181–183).

#### 4.2.2 Golgi protein 73

Golgi protein 73 is a transmembrane protein localized in the Golgi apparatus. Its function is incompletely understood. Golgi protein 73 expression is linked to patients with liver disease, particularly HCC (184, 185). In the serum of HCC patients, Golgi protein 73 concentration is significantly elevated compared to patients with liver cirrhosis (186). Whether Golgi protein 73 alone is superior to AFP in detecting HCC and discrimination from liver cirrhosis is controversial (186, 187). However, combining both markers has improved the detection of early HCC and discrimination from liver cirrhosis (187).

#### 4.2.3 Annexin 2

Annexin 2 is a calcium-dependent, phospholipid-binding protein linked to cell mobility and protein interaction with the actin cytoskeleton. Recent studies reported that Annexins, including Annexin 2, can interfere with immune functions and induce tolerance (188). Interestingly, Annexin 2 is upregulated in HCC and can indicate tumor malignancy (189). Annexin 2 also showed better sensitivity and specificity than AFP to detect early HCC (190). Thus, Annexin 2 might be a helpful marker for early tumor detection (189), although more detailed studies are needed to estimate the potential of Annexin 2 for clinical application.

#### 4.2.4 Osteopontin

Osteopontin (OPN) is an extracellular matrix (ECM) protein whose elevation is associated with tumor invasion, proliferation, and metastasis in several cancers (191). Using tissue microarrays, Desert et al. analyzed 366 samples from patients with normal liver, cirrhosis, dysplastic nodules, or HCC. They show that OPN increases in expression during hepatocarcinogenesis (192). Wu et al. showed in a recent study that OPN induces JAK2/STAT3/NOX1-mediated ROS production, leading to hepatocellular carcinoma progression (193). Several phase 2 studies show osteopontin has diagnostic (194, 195) and prognostic potential (196, 197) as a biomarker.

#### 4.2.5 Midkine

Midkine, also known as neurite growth-promoting factor 2 (NEGF2), is a secreted protein that functions as a cytokine and growth factor and mediates its signal through proteoglycan and non-proteoglycan receptors on the cell surface (198–201). Midkine enhances the angiogenic and proliferative activities of cancer cells. Expression of midkine (mRNA and protein expression) is increased in several cancers, including HCC. Thus, Midkine can serve as a biomarker in HCC, as shown in phase 3 trials (202–205). Midkine is mainly overexpressed in AFP-negative patients, so it increases detection rates of HCC (205).

#### 4.2.6 Dickkopf-1

The glycoprotein Dickkopf-1 (DKK-1), expressed mainly in the placenta and embryonic tissues, is an antagonist of the Wnt/β-catenin signaling pathway and is elevated in several cancer types. DKK-1 shows higher efficacy for detecting HCC than AFP in phase 4 trials (206–208), but midkine is more precise than DKK-1 in cirrhotic HCV patients (209). A combination of Golgi protein 73, AFP, and Dickkopf-1 increases the sensitivity and specificity of HCC detection (210).

#### 4.2.7 Squamous cell carcinoma antigen 1 and 2

Squamous Cell Carcinoma Antigen (SCCA) consists of two proteins, SCCA-1 and SCCA-2, which are serine protease inhibitors. Several studies investigated the diagnostic value of SCCA and its immune complex SCCA-IgM in HCC. A recent phase 4 trial with 203 cirrhotic patients revealed that patients with HCC have higher levels of SCCA-IgM than those without it during a five-year follow-up (211). Another phase 4 trial with 91 patients showed that SCCA-IgM also offers predictive value (212). But gender differences have to be considered, as low levels of SCCA-IgM after transarterial embolization indicate more prolonged survival in males and shorter in females (213). SCCA and SCCA-IgM show moderate diagnostic accuracy in several meta-analyses (214–216). Combination with AFP increases prognostic value significantly (214).

#### 4.2.8 Alpha-l-fucosidase

Alpha-l-fucosidase (AFU) is a lysosomal enzyme that is present in low concentrations in human cells, blood, and body fluid and hydrolyzes fucose-containing sugars. Its activity is increased in the serum and tissue of HCC patients. Still, it is not specific to HCC, as high levels are also found in patients with diabetes, pancreatitis, and hypothyroidism (217). A retrospective phase 3 study with 280 HCC patients due to HBV B AFU shows good early detection prosperities of HCC (218), but midkine is a more sensitive predictor than AFU in HCC due to HCV (209). The combination of AFU with AFP raises sensitivity and specificity, especially in hepatitis-negative patients (219), but is worse than AFP alone in patients with HCC due to HBV (220).

**Conclusion:** The major advantage of protein biomarkers is that the detection is easy to perform, less error-prone, and inexpensive. Results can be obtained quickly and without complex equipment. Therefore, protein biomarkers are optimal for clinical use. The major disadvantage of protein biomarkers is that tumors can escape from detection due to individual differences in the protein expression pattern. However, this can be overcome by using defined combinations of different biomarkers. In the future, defined combinations of protein biomarkers with other types of markers, e.g., circulating nuclear acid and CTCs, may improve detection specificity and sensitivity as well as the prognosis of HCC.

### 4.3 Exosomes

**Figure 5:**
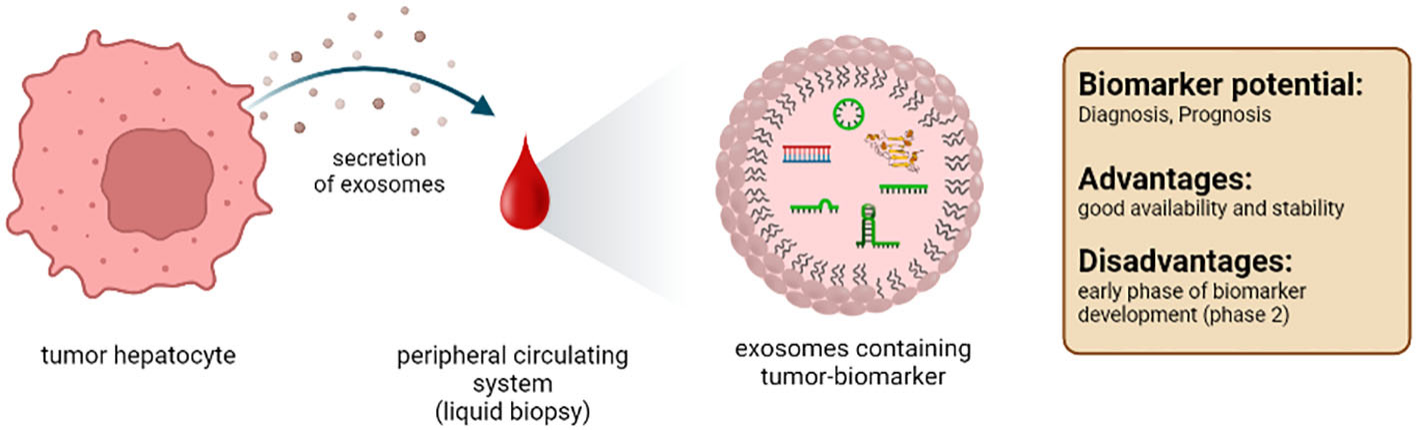
Characteristics of the biomarker class exosomes from a liquid biopsy in patients with HCC.

Exosomes are extracellular vesicles with a diameter of 30-200nm. They are formed in the endosomal compartment of eukaryotic cells. Exosomes are specifically released, facilitating intracellular transport processes, and enabling communication between cells. Their content consists of various components, such as proteins and nucleic acids (**Figure 5**). Therefore, exosomes give detailed information about the secreting cell or tissue (221). In the liver, mainly hepatocytes, immune cells, and non-parenchymal liver cells release exosomes (222). It should be emphasized that the administration of antibiotics from the subgroup of fluocinolones, especially ciprofloxacin, can increase the secretion of exosomes (223). Furthermore, it must be admitted that studies on exosomes as markers are still in the early stages.

#### 4.3.1 Exosomal lipids and proteins

Exosomal membranes are composed of lipids characteristic for different tissue, including tumors. To date, mainly *in vitro* data are available on the potential role of exosomal lipids as biomarkers for HCC (224, 225). Thus, further analysis must show whether exosomal lipids are suitable biomarkers *in vivo*.

Exosomes contain macromolecules, e.g., proteins protected from extracellular degradation processes. Therefore, the exosomal content is potentially interesting for tumor detection and prognosis. One study identified an HCC-specific exosomal protein profile that included CD44, cell division cycle 42 (CDC42), RAS related protein (RRAS), MET, G protein subunit alpha 13 (GNA13), metalloproteinase domain 1 (ADAM1), GNAS complex locus (GNAS), eukaryotic translation initiation factor 4A3 (EIF4A3) and S100 family proteins (226). An additional study detected HSP70, Hsp90, glypican 3, and the well-established marker AFP specific to HCC (227). However, whether exosome-derived protein panels are more valid biomarkers than freely circulating proteins needs to be analyzed in clinical studies, comparing both parameters according to the purification method, the stability, and thus the robustness of the analysis.

Probably the closest study to a clinical application is the HCC EV ECG score, an extracellular vesicle-based protein assay for detecting early-stage hepatocellular carcinoma (228). Here, exosomes isolated from plasma are analyzed for surface expression of EpCAM, CD63, CD147, CD63, and GPC3. The analysis of this panel of surface markers results in a sensitivity of 91% and a specificity of 90% in the early detection of HCC. Thus, this score could complement current monitoring methods and improve patient outcomes.

#### 4.3.2 Exosomal nucleic acids

In addition to proteins, exosomes also contain nucleic acids, e.g., DNA and RNA. Our knowledge of exosomal DNA as biomarkers for HCC is limited to date. Exosome-derived DNA is protected against degradation; it is of high molecular weight and, therefore, suitable as an HCC biomarker (56, 229). More intensively than DNA, exosome-derived RNA has been explored as a potential biomarker for HCC (230, 231). Several types of RNA can be found in exosomes that are protected from degradation by the exosomal membrane and thus have a significantly longer half-life than free RNAs: mRNA, circular RNA, miRNA, and lncRNA. These RNA species, alone or in combination with other exosomal components, represent interesting biomarkers for HCC. For example, RAB11A mRNA was present in exosomes purified from the serum of patients with HCC. Combined with exosomal lncRNA-RP11-513115.6 and miR-1262, it turned out to be an effective biomarker with high sensitivity and specificity in distinguishing patients with HCC from patients with chronic hepatitis C virus infection (232). In addition, exosomal circular RNAs are particularly interesting for predicting the prognosis of HCC. Many circular RNAs are abundant and stable in exosomes derived from patients with HCC, such as hsa_circ_0088030 (circPTGR1), Cerebellar degeneration-related protein 1 antisense RNA (*Cdr1*), and circDB. These circular RNAs promote cancer cell proliferation and metastasis and are indicators of aggressive tumors with poor prognosis (233–236). Another RNA species detected in exosomes are miRNAs. Detection of exosomal miRNA-210 and miRNA-224 is specific for HCC. Both miRNAs promote angiogenesis and enhance the proliferation and invasion of the tumor (237, 238). Exosomal lncRNAs are also potentially significant for the HCC diagnosis. For example, exosomal-derived lnc-FAM72D-3 and lnc-EPC1-4 levels are significantly increased in the serum of patients with HCC (239, 240).

Overall, exosomes contain similar biomarkers compared to those detected in the blood. The vesicle protects the marker molecules from damage and degradation. This is particularly important for nucleic acids as they are targets for degradation in the blood. Further studies must confirm that exosomes and their contents are suitable biomarkers in HCC.

**Conclusion:** Exosomes contain a multitude of important information about the cells from which they originate. Their lipid composition, protein, and nucleic acid content are like a fingerprint of the (tumor) cell from which they originate. However, the purification of these small extracellular vesicles and their analysis are complex and require sophisticated equipment. The quality of the isolation of the inter-exosomal proteins or nucleic acids is highly dependent on the methodology, especially since standardized protocols are not yet available.

Several phase 2 trials show promising results for diagnostic and prognostic biomarker usage (82, 241–243). We need more international studies to prove the importance of exosomes as tumor markers, as most studies are from Asia. From a scientific and clinical point of view, exosomes seem to fulfill all the conditions for an ideal marker (**Table 1**).

### 4.4 Circulating tumor cells

**Figure 6:**
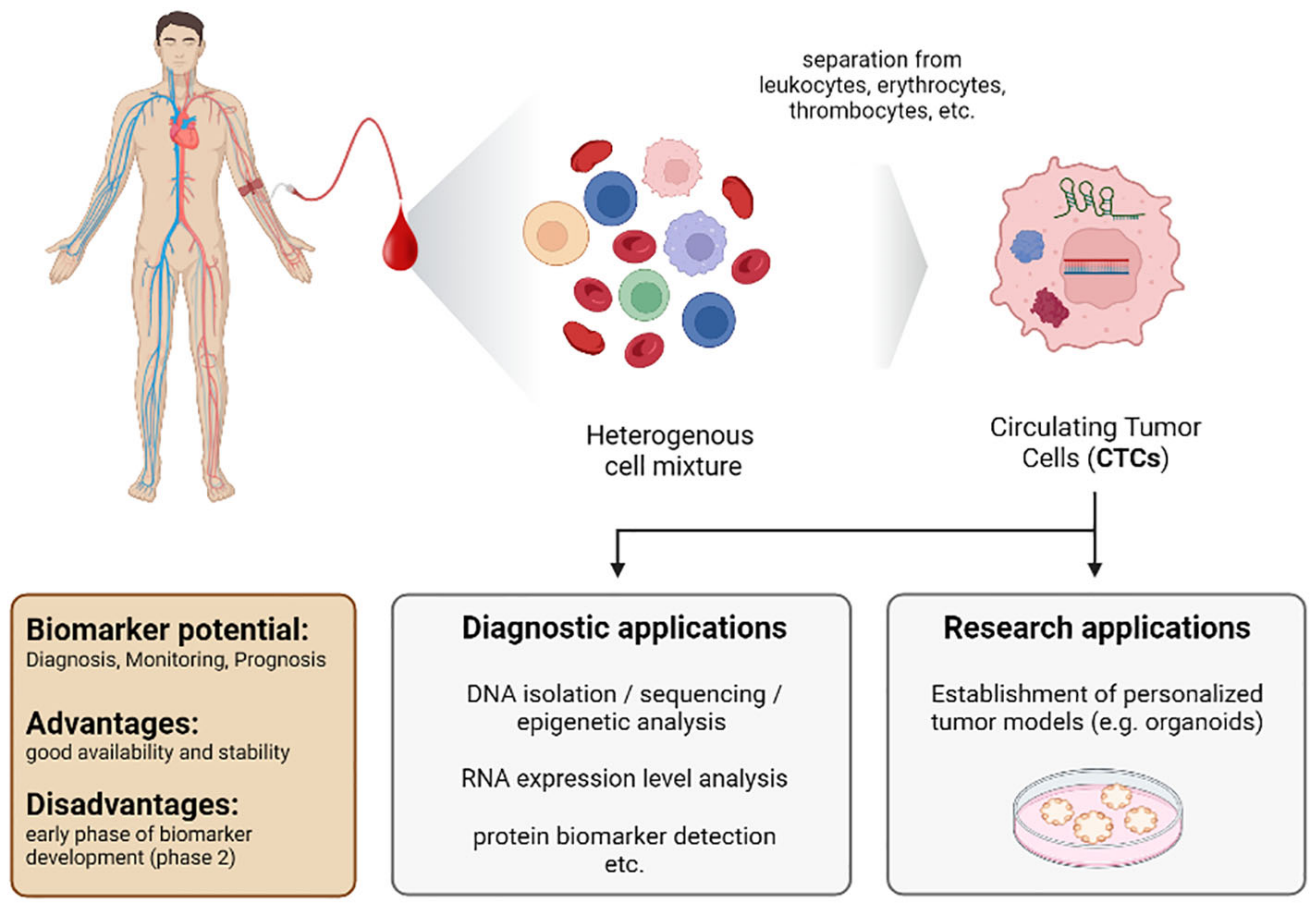
Characteristics of the biomarker class circulating tumor cells (CTCs) from a liquid biopsy in patients with HCC.

In 1869, Ashworth detected “cells similar to those in the tumours” in the blood of a patient with a metastatic tumor (244). This was the first description of CTCs and the first detection of tumors by blood analysis. However, at this time, reliable detection and identification of these cells were a nearly unsolvable challenge. Furthermore, in 1895, x-ray imaging by Roentgen was developed as a novel diagnostic tool. Thus, scientists primarily focused on this new imaging method, and the idea of using CTCs to detect tumors was put on hold. Today we know that imaging tools have their limitations, and additional strategies for early detection and prognosis are urgently needed. Therefore, CTCs again became a focus as biomarkers, especially since detection, isolation, and analysis to investigate CTCs have made tremendous progress (**Figure 6**).

CTCs are cancer cells that circulate in the blood upon being shed off from the tumor. The genetic information of these cells indicates mutations and, therefore, contains hints on sensitivity and resistance towards therapy. In addition, CTCs can be used to form organoids, which serve as personalized tumor models to analyze cell signaling and mimic therapeutic approaches *in vitro* (245–249).

Nevertheless, the isolation and analysis of these cells are still challenging despite novel techniques. Once cells detach from the extracellular matrix, apoptotic cell death, anoikis, is induced in most cells (250). Thus, most tumor cells die within a few hours upon shedding, resulting in a low frequency of CTCs (251, 252). In the blood of patients with metastatic tumors, there is approximately one CTC per 1x10^9^ cells (253). This low frequency makes identification and isolation extraordinarily challenging and asks for strict definitions regarding identification.

The CellSearch™ definition is considered to be the current state-of-the-art standard. This definition states that a CTC is a circulating nucleated cell larger than 4µm, expressing the epithelial cell adhesion molecule (EpCAM) and cytokeratins 8, 18, and 19. To separate CTCs from immune cells, the CTC has to be negative for the leukocyte-specific antigen CD45 (254). However, only about one-third of CTCs derived from HCC patients are positive for EpCAM and cytokeratins. Thus, the application of CellSearch™ criteria is unsuitable for all subgroups of HCC (255, 256). Another concern is that the level of CTCs correlates with tumor burden. Therefore, sensitivity in the early stage of the disease might be low (252).

Nevertheless, several studies using CellSearch™ showed interesting results for CTCs as diagnostic and prognostic markers. One study analyzed patients with HCC before and one month after liver resection. The number of CTCs was a reliable diagnostic and prognostic marker, indicating the reoccurrence of the tumor (257). Another study using CellSearch™ criteria demonstrated the frequent presence of CTCs in patients with intermediate and advanced HCC (258). Further CellSearch™-based studies showed that the appearance of EpCAM-positive CTCs in HCC could be used to predict recurrence and is associated with poor prognosis (259, 260).

In one study, the nanofiltration technique CanPatrol™ was used to identify clusters of CTCs and white blood cells. Patients exhibiting those clusters show significantly shorter disease-free survival and overall survival (261). Another study using CanPatrol™ shows that a high percentage of mesenchymal CTCs are closely related to the expression of CK19, which is associated with a poor prognosis in HCC patients (262). Qi et al. use CanPatrol™ combined with an RNA-ISH assay to enrich and classify CTCs from patients with HCC. In this study, a slightly increased percentage (≥2%) of mesenchymal CTCs before resection was shown to be a predictive factor for early tumor recurrence (263).

Interestingly, other identification criteria based on less strict definitions also revealed good results for detecting HCC. One study reported that CTCs identified only based on their morphology were associated with shorter survival in HCC (264). In another study, erythrocytes and CD45^+^ immune cells were depleted from the pool of circulating cells. Afterward, the expression pattern of the leftover cells was monitored by polymerase chain reaction (PCR) using differential expression of EpCAM, CD90, CD133, and CK19 as identifiers for CTCs. Of note, this analysis revealed a sensitivity of 72.5% and a very high specificity of 95% to detect HCC in healthy donors and discriminate HCC from chronic HBV infection and benign hepatic lesions. Using AFP as a biomarker displayed a sensitivity of 57% and a specificity of 90%. Of clinical relevance, this approach also performed well in patients with early-stage HCC and showed a sensitivity of 71.8% and a specificity of 95%. Thus, this method could be a risk prediction and treatment surveillance tool enabling early decision-making to adjust effective antitumor strategies (265). Results of an interesting recent study showed that the detection of CTCs in patients who have undergone LTx allows early prediction of recurrence. Therefore, serial CTC detection may be helpful in the postoperative monitoring of HCC recurrence. However, it has to be considered that the study design is limited due to its small patient cohort, relatively short follow-up of the course, and its single-center design. Therefore, further clinical studies have to follow (243).

**Conclusion:** The major advantage of CTCs as biomarkers is that these cells contain all the information about the tumor. Furthermore, looking for markers of epithelial-mesenchymal transition determines metastasis formation and aggressiveness of a tumor. Therefore, the analysis of CTCs will advance our understanding of the biology of metastatic diseases and the development of treatment strategies. The required blood volumes are also practical and do not exceed 10ml. The disadvantage of CTCs as biomarkers is that purification and detection are complicated, time-consuming, and costly. The necessary technical equipment may not be available in all clinical settings. Thus, further improvement in the detection and isolation of CTCs is required to use them routinely as biomarkers in the clinic. On the one hand, PCR-based methods might address the challenges in detecting and isolating CTCs and lead to less time-consuming and cost-intensive investigations. On the other hand, such strategies entirely depend on the quality and definition of target transcripts for the detection and staging of the tumor. It is important to note that the control groups in most studies of CTCs were small. In addition, most studies were conducted in East Asia (China, Japan, and Taiwan). Thus, ethnic differences cannot be excluded. Most studies are in phase 2 of biomarker development (257, 261, 263, 265–268) (**Table 1**). Prospective studies are needed.

## 5 Final conclusion

HCC is one of the most common tumor diseases with rising incidence and high mortality. Management of HCC especially requires early diagnosis and therapy. Therefore, we need reliable, valid, and objective biomarkers for screening, diagnosis, disease monitoring, prognosis, predicting response to therapy, and treatment safety. Identification of novel non-invasive biomarkers for HCC has become the focus of research. There is an urgent need to define circulating markers that can replace invasive methods like liver biopsies and provide additional information about the tumor. These markers would enable more personalized medicine, including the prediction of therapeutic response. CTCs, ctDNA, circulating RNA, and exosomes are attractive candidates for liquid biopsy since they fulfill many essential characteristics of an ideal biomarker.

A big step towards ideal biomarkers is certainly the analysis of ctDNAs. ctDNA is stable and provides epigenetic and genetic data on the tumor. Detection of mutated DNA and methylation profiling is suitable for early detection of HCC and estimation of prognosis. However, NGS and methylation profiling is complicated and time-consuming. In addition, the different studies often identify completely different markers. It is difficult to understand why there are often no similarities between the markers identified in the studies. One reason is certainly a high heterogeneity between individual tumors. However, this cannot explain the lack of common markers in the studies. The differences must also be due to different study conditions. Here we need standardized and validated protocols for ctDNA purification and analysis. The quality of the samples is the key to a valid statement on the quality of ctDNA as a potential tumor marker.

miRNA and lncRNAs have shifted into the focus of cancer research. So far, more than 70 miRNAs have been proposed as potential biomarkers for HCC, and more and more lncRNA markers have been identified. Most remarkable results for the detection and prognosis of HCC were obtained using well-defined marker panels. These combinations of different RNAs showed very good results in sensitivity and specificity. Other combinations, e.g., with protein markers, are also possible. However, there are often entirely different marker panels, and the studies are not able to confirm the panels from other studies. Again, the quality of the samples plays a crucial role. We need standardized protocols to be used in studies worldwide. This is the only way to identify unique markers that can be used universally. This is true for HCC, but also all other tumors.

Proteins, including AFP, are the best-characterized biomarkers. They do not need elaborate purification and detection methods. Thus, they are optimal to be used in clinical routine. However, they often lack specificity. This disadvantage can be overcome using a combination of different markers or a panel of protein markers and other parameters. A very good example for the combination of several markers is the GALAD score. The score shows that combining three protein markers that can be easily determined in routine clinical practice (AFP, AFP-L3, and DCP) with patient metadata can significantly improve the predictive value. The combination of the three protein markers significantly increases prediction sensitivity but decreases specificity. However, adding simple patient metadata such as age and gender to the protein markers significantly improves both sensitivity and specificity. However, other combinations of tumor markers are also conceivable. The combination of protein biomarkers and nucleic acid markers also shows initial success and leads to improved sensitivity and specificity in prediction. Again, it must be said that more studies from Europe and North America are important to support the findings and reconcile ethnical differences.

Even though fewer data are currently available, analysis of exosomes could provide novel options to detect and understand the development of HCC. Exosomes contain unique functional information, e.g., about interactions between cancer cells and distant cells or the tumor microenvironment. Thus, they are important to gain more insights into tumor physiology. There is certainly a long way to go for exosomes as biomarkers until clinical application. However, they represent a tool with many advantages. Many macromolecules are already degraded in the body or during the purification process. In exosomes, they are protected. Thus, errors can be avoided, and differences due to purification can be prevented. This could help to solve the challenges described above for nucleic acid biomarkers.

CTCs are the carbon copy of the tumor itself. On the one hand, they can provide genetic information about mutations and epigenetic alterations of the tumor. Moreover, CTCs exhibit the transcriptome and proteome of the cancer of origin. Thus, they fulfill all criteria of an ultimate biomarker. On the other hand, they occur sparsely in blood, and there are no optimal/general surface markers for HCC-derived CTCs. Thus, purification and identification are very challenging and costly. Nevertheless, the blood volumes required for purification are practical (not exceeding 10ml). Regardless, new appropriate purification and analysis methods must be developed to routinely use these biomarkers in the clinic. Additionally, it is unfortunate to note that most studies on CTCs and other tumor biomarkers were conducted in East Asia. We need more studies in the Western industrialized nations since it is precisely here that an increase in HCC is apparent. This is primarily due to lifestyle and associated malnutrition.

We are on the right track in identifying new biomarkers, but we need more and better studies. Since HCC is a worldwide challenge, we need international studies to consider ethnical differences. To establish reliable universal markers, we need standardized and validated purification, storage, and processing protocols for the corresponding macromolecules or CTCs. This is the only way to identify and develop new good tumor markers.

After developing and validating novel biomarkers, the final step has to be their integration into the clinical routine. The novel liquid biopsy-based tools will not replace the established methods but will supplement them to optimize patient care. These markers are non-invasive or minimally invasive and, therefore, easier to implement, as only small volumes of biological material (e.g., blood) are required. As mentioned before, the challenge is certainly the purification and analysis of the samples. However, there are great technological advances that help us to overcome these obstacles. Therefore, we can look optimistically into the future and assume that we will have significantly more and better biomarkers for HCC in the near future.

## Author contributions

Conceptualization: MM, SoS and KG. Investigation: SoS and KG. Original draft preparation: MM, KG and SoS. Review and editing: MM, KG, CK, SoS, AK, StS, DT, HT. Table preparation: SoS, KG, BV, KN, NE. Visualization: KG, SoS and DT. Supervision: MM. All authors contributed to the article and approved the submitted version.

## Acknowledgments

Pictures have been created with BioRender.com.

## Conflict of interest

The authors declare that the research was conducted in the absence of any commercial or financial relationships that could be construed as a potential conflict of interest.

## Publisher’s note

All claims expressed in this article are solely those of the authors and do not necessarily represent those of their affiliated organizations, or those of the publisher, the editors and the reviewers. Any product that may be evaluated in this article, or claim that may be made by its manufacturer, is not guaranteed or endorsed by the publisher.
